# Foot and ankle characteristics associated with falls in adults with established rheumatoid arthritis: a cross-sectional study

**DOI:** 10.1186/s12891-016-0888-z

**Published:** 2016-01-13

**Authors:** Angela Brenton-Rule, Nicola Dalbeth, Hylton B. Menz, Sandra Bassett, Keith Rome

**Affiliations:** AUT University, Private Bag 92006, Auckland, 1142 New Zealand; University of Auckland, Private Bag 92-019, Auckland, New Zealand; La Trobe University, Melbourne, Victoria 3086 Australia

**Keywords:** Rheumatoid arthritis, Falls, Fall risk factors, Feet, Balance, Disability, Impairment

## Abstract

**Background:**

People with rheumatoid arthritis (RA) have an increased risk of falls. The foot is a common site of pathology in RA and foot problems are reported in up to 90 % of patients with established disease. The aim of this study was to determine whether foot and ankle characteristics are associated with falls in people with RA.

**Methods:**

Adults with RA were recruited from rheumatology outpatient clinics in Auckland, New Zealand. Participants reported whether they had fallen in the preceding year, and the number of falls. Clinical characteristics, common fall risk factors, and foot and ankle variables were measured. Univariate parametric and non-parametric analysis compared fallers and non-fallers on all variables to determine significant differences. Logistic regression analysis identified variables independently associated with falls.

**Results:**

Two hundred and one participants were prospectively recruited. At least one fall in the preceding 12-months was reported by 119 (59 %) participants. Univariate analysis showed that fallers had significantly longer mean disease duration, more co-morbid conditions, an increase in lower limb tender joints, higher midfoot peak plantar pressures and were more likely to have a history of vascular disease than non-fallers. Fallers also reported greater difficulty with activities of daily living, increased fear of falling and greater self-reported foot impairment. Logistic regression analysis revealed that increased midfoot peak plantar pressures (odds ratio (OR) 1.12 [for each 20 kPa increase], 95 % confidence interval (CI) 1.00-1.25), self-reported foot impairment (OR 1.17 [for each three point increase], 95 % CI 1.05-1.31) and history of vascular disease (OR 3.22, 95 % CI 1.17-8.88) were independently associated with a fall in the preceding 12 months.

**Conclusions:**

Elevated midfoot peak plantar pressures, self-reported foot impairment and vascular disease are associated with falls in people with RA. Assessment of foot deformity, foot function and self-reported foot impairment may be of benefit when considering falls prevention strategies in people with RA.

**Trial registration:**

Australia New Zealand Clinical Trial Registry (trial ACTRN12612000597897).

## Background

Falls represent an important burden to healthcare resources worldwide and treatment of fall related injuries accounts for a significant proportion of healthcare spending [1, 2]. For individuals, loss of confidence and independence as a consequence of falling can significantly reduce quality of life. Education regarding fall risk and prevention is fundamental to health and well-being in older adults [3].

People with rheumatoid arthritis (RA) are at greater risk of falling than healthy older adults [4]. RA is a chronic, inflammatory, autoimmune disease characterised by systemic inflammation, persistent synovitis and progressive articular destruction [5]. Most synovial joints can be affected, however, the peripheral joints are predominantly involved, most often the small joints of the hands and feet, and usually in a symmetrical distribution [6]. Previous studies in people with RA have reported falls incidence ranging from 10 to 54 % [4, 7–12]. Fall risk factors common to older people have been identified in people with RA [4, 9–11, 13]. These include history of a previous fall [4, 11], fear of falling [4], impaired general health [13], number of co-morbid conditions [10], fatigue and dizziness [4] and antihypertensive medication [9]. Fall risk factors which may be RA disease-specific have also been reported including activity limitation as measured by the Health Assessment Questionnaire (HAQ) [4, 13], tender joint count [4, 13], swollen joint count [9], 28 joint Disease Activity Score (DAS28) [4], pain intensity [4, 11], number of medications [4, 7], use of corticosteroids, psychotropic medications [4] and antidepressants [7]. Decreased lower extremity muscle strength [4] and impaired standing balance [4, 9] have also been associated with falls in people with RA.

The foot is a common site of pathology in RA, with many patients reporting foot symptoms at initial diagnosis, and as many as 90 % of people with established RA report current foot problems [6]. Postural stability is also decreased in people with RA compared to the non-RA population. As a result, people with RA have difficulty maintaining postural control when undertaking everyday activities [14]. Several studies in healthy older adult populations have identified foot and ankle characteristics which may impair balance and increase the risk of falling [15–21]. The foot and ankle characteristics related to fall risk factors in older adults include lesser toe deformity [16], reduced ankle range of motion [21], severe bunion deformity [21], pes planovalgus foot-type [15], reduced plantar sensitivity [15, 21], decreased toe strength [16, 21], disabling foot pain [15, 17, 21], slower gait speed [22] and increased peak plantar pressures and pressure–time integrals [17].

There is a dearth of evidence regarding foot and ankle characteristics and falls in people with RA. The aim of this study was therefore to determine the foot and ankle characteristics associated with falls in people with RA. This paper reports the findings of a cross-sectional study in adults with established disease. We compared fallers and non-fallers, according to falls experienced in the preceding 12 months, on a range of foot and ankle measures and common falls risk factors.

## Methods

### Participants

We recruited participants from rheumatology outpatient clinics in the wider Auckland region of the North Island, New Zealand. All participants were English speaking adults (18 years and older) with RA according to the 2010 American College of Rheumatology/European League Against Rheumatism classification criteria [23]. Participants were excluded if they were non-ambulatory or unable to attend a study visit at a specified clinic or research facility. Participants were also excluded if they were unable to read and understand the information sheet or sign the consent form. Based on a previous falls study involving 176 older adults [21], an a priori sample size calculation, based on a 15 % dropout rate, 80 % power, and a significance level of 5 %, indicated that 200 participants were needed. The study was conducted with the approval of the Northern X Regional Ethics Committee (reference NTX/11/12/114) and the Auckland University of Technology Ethics Committee (reference 12/47).

### Data collection

Participant characteristics were recorded including age, gender, ethnicity and body mass index (BMI). Medical records were accessed to confirm RA disease type, disease duration, erosive foot disease (as evidenced by radiographs), previous foot surgery, blood tests, medications and co-morbid conditions. All clinical assessments were completed at a single baseline study visit by one researcher (ABR). The researcher is a New Zealand registered podiatrist with five years of clinical practice experience and postgraduate research training. RA disease activity was assessed by the number of tender and swollen joints and calculation of the four variable DAS28-CRP [24]. Activity limitation was assessed using the HAQ-II [25]. Participants also completed the Short Falls Efficacy Scale-International (Short FES-I) and the Foot Impact Scale (FIS) questionnaires. The Short FES-I consists of seven questions which assess fear of falling related to a range of daily activities [26]. The FIS comprises two subscales for impairment/footwear (FIS_IF;_ range 0–21) and activities/participation (FIS_AP;_ range 0–30) [27]. An elevated FIS_IF_ or FIS_AP_ score indicates greater foot impairment or activity limitation respectively, and a three point change is considered to be clinically relevant [28, 29]. In accordance with a previous study [28], scores of ≤6 were considered mild, from 7 to 13 were considered moderate, and ≥14 were considered severe for FIS_IF_. For FIS_AP_, scores ≤9 were considered mild, 10–19 were considered moderate, and ≥20 were considered severe. Global pain in the past week and current patient global assessment of disease activity were recorded using a 100 mm visual analogue scale (VAS). A score of 0 mm on the pain scale indicated ‘No Pain’ and 100 mm indicated ‘Severe Pain’. On the patient global assessment scale, a score of 0 mm indicated ‘Very Well’ and 100 mm indicated ‘Very Unwell’. Participants were asked whether they had ever visited a podiatrist, and if so, the frequency of podiatric care received.

Assessment of foot and ankle characteristics included foot problem score (FPS), plantar sensation, foot and ankle muscle strength, ankle range of motion (ROM), toe strength, gait speed, peak plantar pressure and postural stability. The FPS is calculated based on a grading of hallux valgus [30], lesser toe deformity, hyperkeratotic lesions, abnormal bony prominences and VAS foot pain [19]. Plantar sensation at six sites [31] was assessed as fine touch, using a 10 g monofilament, and as vibration perception threshold, using a biothesiometer [32, 33]. Foot and ankle muscle strength (dorsiflexion, plantarflexion, inversion, eversion) was assessed using a hand-held dynamometer (HHD) using the make-test, in which the assessor resists the movement of the muscle [34]. Ankle ROM was assessed by a modified lunge test using an upright acrylic sheet to measure the angle formed by the head of the fibula and the lateral malleolus in relation to the horizontal plane [35]. Toe strength was assessed using the Paper Grip Test whereby patients are instructed to grip a business card while an assessor attempts to pull it out from underneath the hallux and lesser toes. [21]. Gait speed was assessed using the 6-m walk test [19]. Participants were tested in the footwear which they wore to the study visit and instructed to walk at their usual speed. Peak plantar pressure was recorded for both feet using a TekScan MatScan® (TekScan Inc, South Boston, USA) pressure mat [17]. The foot was divided into forefoot, midfoot and rearfoot for analysis. The forefoot was defined as 50 % of foot length, the midfoot was 19 % and the rearfoot was 31 % of foot length as previously reported [36]. Participants were tested in bare feet using a 2-step protocol [17]. The MatScan® was also used to assess postural stability which was measured as postural sway (oscillations around the centre-of-mass) in the anterior-posterior (AP) and medial-lateral (ML) directions, during quiet standing [37]. Participants were tested in bare feet with eyes open for 30 s in quiet bipedal standing. Extrinsic fall risk factors were also recorded including footwear worn to the study visit, the use of visual aids and use of assistive devices. Footwear type was identified using a list of 17 footwear styles adapted from a previous study [38] and grouped into good, average and poor for reporting [39].

### Falls data ascertainment

The primary outcome measure for the study was fall history. The number of falls experienced during the preceding 12 months was recorded as “no falls”, “one fall” or “more than one fall”. The Prevention of Falls Network Europe (ProFaNE) definition of “an event that results in a person coming to rest unintentionally on the ground or other lower level” was used to identify falls [2]. We modified the fall definition by adding, “not as a result of a major intrinsic event or an overwhelming hazard”, as per previous study [21] to exclude falls which were the result of syncope or an external force. Falls data were obtained by providing the participant with the fall definition and then asking, “In the past 12 months, have you had any fall including a slip or trip in which you lost your balance and landed on the floor or ground or lower level?” Falls history was recorded at the end of the study visit following completion of all clinical and foot and ankle measures.

### Statistical analysis

Participants were grouped as ‘fallers’ or ‘non-fallers’ based on the 12-month fall history. Comparisons between groups were made using independent samples student’s *t* tests for normally distributed variables and Mann–Whitney *U* tests for skewed data. Chi-square tests of trend were used as appropriate to examine differences between groups on categorical variables. To identify factors independently associated with a history of falls, a logistic regression model was created using falls in the preceding 12 months as the dependent variable. A limited number of predictor variables were selected based on statistical significance of *P* < 0.15 on univariate analysis. Where multicollinearity (*r* ≥ 0.7) was present, the variable with the lowest *P* value, or of greatest clinical relevance, was retained. Multivariate binary logistic regression analyses were conducted including the selected predictor variables adjusting for age. The backward elimination method was used to remove the variable with the highest *P*-value, in a stepwise approach, until all remaining variables were significant at *P* < 0.05. Statistical analysis was performed using Statistical Package for Social Sciences V22.0 (IBM Corp., New York, USA).

## Results

### Participant characteristics

Two-hundred and one participants were recruited into the study and completed the baseline study visit. Baseline clinical characteristics and common fall risk factors for the participants are presented in Table 1. Participants were predominantly female (*n* = 150, 75 %). The mean (SD) age of female participants was 63 (11) years and male participants was 68 (10) years. Most of the participants (*n* = 161, 80 %) identified as European and had well established RA, with mean (SD) disease duration 16 (14) years. Sixty-nine percent had co-morbid conditions with hypertension (*n* = 73, 36 %) and osteoporosis (*n* = 39, 19 %) being the most common. Most participants (*n* = 175, 87 %) were taking non-biologic disease modifying anti-rheumatic drugs (DMARDs) and over one third (*n* = 74, 37 %) were taking prednisone. Anti-hypertensive medication (*n* = 91, 45 %) was also common. On the day of the study visit, participants had moderate disease activity and reported mild to moderate disability, with mean (SD) scores: DAS28 3.38 (1.26) and HAQ-II 0.89 (0.62).

**Table 1:** Clinical characteristics and common fall risk factors (*n* = 201). Data presented as mean (SD) unless specified

Age	64.7 (11)
Women, n (%)	150 (75)
European, n (%)	161 (80)
Body Mass Index, Kg/m^2^	27.8 (5.3)
Disease duration, years	15.9 (13.6)
Disease type, n (%)	
Rheumatoid factor positive	162 (81)
Anti-CCP antibodies positive	105 (76)
Seronegative	26 (13)
RA medications, n (%)	
DMARD monotherapy	71 (35)
Combination DMARD therapy (≥2 DMARDs)	104 (52)
Biologics	33 (16)
Prednisone	74 (37)
Other medications, n (%)	
Opiates	18 (9)
Anti-platelets	44 (22)
Anti-coagulants	11 (6)
Anti-hypertensive	91 (45)
Hypoglycaemias	16 (8)
Psychotropics	37 (18)
Number of medications	4.1 (2.0)
Number of co-morbidities	1.2 (1)
Co-morbid conditions, n (%)	
Hypertension	73 (36)
Other vascular disease	29 (14)
Diabetes Mellitus	18 (9)
Parkinson’s disease	2 (1)
Osteoporosis	39 (19)
Depression or bipolar disorder	16 (8)
Patient self-reported pain (VAS 0–100), mm	39 (27)
Patient global (VAS 0–100), mm	36 (26)
Tender joint count (total)	11 (12)
Swollen joint count (total)	5 (7)
DAS28-CRP score	3.38 (1.26)
Short FES-I score (7–28)	12 (5)
HAQ-II score	0.89 (0.62)
Wears glasses or contact lenses, n (%)	181 (90)
Uses assistive device, n (%)	57 (28)

*VAS* visual analogue scale, *DAS* disease activity score, *FES-I* Falls Efficacy Scale-International, *HAQII* Health Assessment Questionnaire

Table 2 describes the foot and ankle characteristics in the entire group. Foot disease was frequently observed; 62 % (*n* = 112) had radiographic erosions in the feet, bunion deformity was present in 65 % (*n* = 130) and 34 % (*n* = 68) had pes planovalgus foot-type. Half of the participants failed the paper grip test for the hallux (*n* = 101, 50 %) and lesser digits (*n* = 102, 50 %) and 43 % (*n* = 85) had a vibration perception threshold greater than 26 mV, indicating presence of neuropathy. Seventy-three percent (*n* = 147) of participants experienced foot pain in the past week, with a mean (SD) of 32 (29) using a 100 mm VAS. On examination, 74 % (*n* = 148) presented with tender and/or swollen lower limb joints. On the day of the study visit, participants reported moderate levels of foot impairment and disability, with mean (SD) score of 10 (5) for impairments/footwear (FIS_IF_) and 15 (9) for activities/participation (FIS_AP_). Just over half of the participants (*n* = 102, 51 %) wore footwear classified as ‘good’ to the study visit. Fifty-three percent (*n* = 107) had previously visited a podiatrist but only 16 % (*n* = 32) received regular podiatry treatment.

**Table 2:** Foot and ankle fall risk factors (*n* = 201). Data presented as mean (SD) unless specified

Foot erosion on radiograph, n (%)	112 (62)
Previous foot surgery, n (%)	29 (21)
Presence of foot pain, n (%)	147 (73)
Foot pain (VAS 0–100), mm	32 (29)
Tender joint count (lower limb)	6.0 (7.2)
Swollen joint count (lower limb)	1.8 (3.4)
Presence of tender and/or swollen joints (lower limb), n (%)	148 (74)
Foot problem score	15 (8)
Pes planovalgus foot-type, n (%)	68 (34)
Presence of bunion deformity, n (%)	130 (65)
Monofilament sites felt (0–12)	10 (3)
Vibration perception threshold ≥ 26 mV, n (%)	85 (43)
Foot muscle strength, N	
Dorsiflexion	63 (33)
Plantarflexion	69 (29)
Inversion	33 (17)
Eversion	30 (15)
Ankle range of motion, degrees	58 (7)
Gait speed, m/s	1.07 (0.3)
Peak plantar pressure, kPa	
Forefoot	310 (66)
Midfoot	113 (62)
Rearfoot	243 (69)
Failed paper grip test hallux, n (%)	101 (50)
Failed paper grip test lesser toes, n (%)	102 (50)
Toe strength hallux, N (%BW)	4.5 (2.5)
Toes strength lessor toes, N (%BW)	2.1 (1.2)
Eyes-open postural sway, mm	
AP direction	20.9 (9.7)
ML direction	14.2 (8.7)
Eyes-closed postural sway, mm	
AP direction	29.4 (12.9)
ML direction	17.4 (9.7)
Has seen a podiatrist before, n (%)	107 (53)
Receives regular podiatry treatment, n (%)	32 (16)
FIS_TOTAL_ score (0–51)	25 (12)
FIS_IF_ subscale score (0–21)	10 (5)
FIS_AP_ subscale score (0–30)	15 (9)
Good footwear type worn to study visit, n (%)	102 (51)
Average footwear type worn to study visit, n (%)	18 (9)
Poor footwear type worn to study visit, n (%)	81 (40)

*VAS* visual analogue scale, *AP* antero-posterior, *ML* medio-lateral, *FIS*_*IF*_ foot impact scale impairment/footwear subscale; *FIS*_*AP*_ foot impact scale activities/participation subscale

### Univariate analysis comparing fallers and non-fallers

Of the 201 participants with RA, 119 (59 %) reported one or more falls in the preceding 12 months. Of those that fell, 46 (39 %) fell once (single-fallers) and 73 (61 %) fell more than once (multiple-fallers). Age (*P* = 0.69) and female sex (*P* = 0.58) were not associated with falls. The results of univariate analysis comparing non-fallers and fallers on other baseline measures are presented in Table 3. Results shown are for comparisons with *P* < 0.05. Fallers had significantly longer mean disease duration (*P* = 0.03), an increase in lower limb tender joints (*P* = 0.04), more co-morbid conditions (*P* = 0.02) and higher midfoot peak plantar pressures (*P* = 0.007). There was a significant difference in HAQ-II score between the groups with fallers reporting greater difficulty with the activities of daily living, compared to non-fallers (*P* = 0.01). Fallers also reported greater fear of falling with significantly higher short FES-I scores (*P* = 0.002) than non-fallers. Foot disability and impairment was greater in fallers compared to non-fallers with significantly higher scores recorded for the activities/participation subscale (FIS_AP_) of the Foot Impact Scale (*P* = 0.002). Compared to non-fallers, those who fell were more likely to have a history of vascular disease including stroke, ischemic heart disease, arrhythmia and peripheral vascular disease (*P* = 0.03).

**Table 3:** Univariate analysis of non-fallers and fallers^a^. Data presented as mean (SD) unless specified

	Non-fallers *n* = 82	Fallers *n* = 119	*P* value
Clinical features			
Disease duration	13.6 (12.8)	17.4 (13.9)	0.03
HAQ-II	0.76 (0.60)	0.98 (0.62)	0.01
Number of co-morbid conditions	0.96 (0.92)	1.33 (1.11)	0.02
Vascular disease, n (%)	6 (7)	23 (19)	0.03
Fear of falling (short FES-I)	11 (5)	13 (5)	0.002
Foot and ankle features			
Tender joint count (lower limb)	4.98 (6.9)	6.63 (7.37)	0.04
Peak plantar pressure midfoot, kPa	100 (44)	122 (71)	0.007
Patient-reported foot impairment (FIS_AP_)	12 (9)	16 (8)	0.002

^a^Comparisons with *P* < 0.05 are shown

*HAQ* Health Assessment Questionnaire, *FES-I* Falls Efficacy Scale-International, *FIS*_*AP*_ Foot Impact Scale Activities/Participation subscale

### Multivariate analysis of predictive risk factors

Multivariate logistic regression analysis identified associations between potential fall risk factors and falls in the preceding 12-months. The results comparing all fallers with non-fallers using logistic regression analyses are shown in Table 4. Seventeen variables, plus age, were entered into the original model including: pes planovalgus foot-type, tender joint count (lower limb), gait speed, foot problem score, hallux strength, ankle range of motion, peak plantar pressure midfoot, eyes closed AP sway, FIS_AP_, disease duration, DAS28-CRP, DMARD monotherapy, combination DMARD therapy, number of medications, number of co-morbid conditions, vascular disease and osteoporosis. To avoid multicollinearity, HAQ-II and FES-I were excluded from the model as they were both highly correlated (R ≥ 0.7) with FIS_AP_. In addition, total tender joint count was excluded as it was highly correlated with tender joint count (lower limb). The final model contained three variables which were independently associated with a fall in the preceding 12-months (*P* < 0.05): history of vascular disease (odds ratio (OR) 3.22, 95 % confidence interval (CI) 1.17-8.88, *P* = 0.024), increased midfoot peak plantar pressure (OR 1.12 [for each 20kPa increase], 95 % CI 1.00-1.25, *P* = 0.046) and self-reported foot impairment (OR 1.17 [for each tress point increase], 95 % CI 1.05-1.31, *P* = 0.005).

**Table 4:** Stepwise logistic regression analyses comparing fallers (*n* = 119) and non-fallers (*n* = 82) on all predictor variables adjusting for age^a^

	Odds ratio (95 % confidence interval)	*P* value
Age	0.99 (0.96-1.02)	0.500
Vascular disease	3.22 (1.17-8.88)	0.024
Peak plantar pressure midfoot	1.12 (1.00-1.25)	0.046
(for each 20kPa increase)		
Foot Impairment FIS_AP_	1.17 (1.05-1.31)	0.005
(for each 3 point increase)		

^a^Associations with *P* < 0.05 are shown

*kPa* = kilopascals, *FIS*_*AP*_ Foot Impact Scale Activities/Participation subscale

Variables included in the model: age, pes planovalgus foot-type, tender joint count (lower limb), gait speed, foot problem score, hallux strength, ankle range of motion, peak plantar pressure midfoot, eyes closed AP sway, FIS_AP_, disease duration, DAS28-CRP, DMARD monotherapy, combination DMARD therapy, number of medications, number of co-morbid conditions, vascular disease and osteoporosis

## Discussion

In this study, 59 % of participants reported at least one fall during the preceding 12-months. This is much higher than the 30 % reported for community dwelling older adults [40] and is consistent with reports that adults with RA are at increased risk of falling compared to the non-RA population [4, 9, 11]. Previous studies in RA populations have identified fall risk factors common to older adults generally, as well as disease-specific fall risk factors. The current study sought to extend our understanding of fall risk in this group through the inclusion of foot and ankle measures known to be associated with falls in healthy older adults.

We found fallers to have higher peak plantar pressures in the midfoot region compared to non-fallers. Midfoot peak plantar pressure was also found to be independently associated with falls in the previous year, with the odds of falling increasing by 12 % for each 20kPa increase in pressure. Increased midfoot plantar pressures have been reported in RA patients with pes planovalgus (flatfoot) deformity, leading to adaptive changes which may compromise stability and increase falls risk compared to healthy controls [41]. Increased pressure at the midfoot is also associated with a pronated foot posture, which has previously been shown to impair postural stability in healthy younger adults [42]. This is the first study to assess plantar pressures as a potential fall risk factor in people with RA. Our results suggest that measurement of plantar pressures, at the midfoot, may be useful in identifying people with RA at increased fall risk. Plantar pressure systems are commonly used in clinical practice to identify areas of high pressure which may compromise tissue viability in patients with high risk foot conditions [43]. Such equipment could also be utilised to identify increased pressures at the midfoot, as part of a fall risk assessment in patients with RA. In clinical settings where pressure analysis equipment is unavailable, the identification of a pes planovalgus foot-type may suffice as an indicator of increased midfoot pressures, particularly in the presence of callosities at the talonavicular joint [41].

In agreement with previous studies [4, 11, 22], self-reported disability and impairment (HAQ-II score) and fear of falling (short FES-I score) were significantly associated with increased risk of falling. Foot-related disability and impairment were also found to be independently associated with a history of falls. Specifically, the odds of falling increased by 17 % for each three point increase on the FIS_AP_ subscale. The FIS was designed specifically to assess the impact of RA disease-related foot involvement in terms of impairment, disability and quality of life [27]. The current findings suggest that the FIS, particularly the activities/participation subscale, may also be useful in identifying and monitoring people with RA with increased risk of falling. Clinical guidelines recommend the use of patient-reported outcome measures (PROMs) in the management of foot health in people with RA [44]. Our findings suggest that PROMs, including the HAQ-II, the Short FES-I, and in particular, the FIS may be also useful in identifying people with RA with increased risk of falling and could be incorporated as part of a regular fall risk assessment.

In the current study, lower limb tender joint count was significantly higher for fallers compared to non-fallers. Lower limb joint count included the knees, ankles and sixteen joints in each foot. Several previous falls studies in RA populations have assessed tender joints as a risk factor for falls, with conflicting results [4, 9, 10, 13]. However, only one study reported findings related to the lower extremities [4]. In this study, Stanmore et al. [4] found that the presence of tender or swollen lower extremity joints was an independent predictor of falls (OR 1.7). However, lower extremity joints included the hips, knees and ankles only [4]. Joint tenderness in RA is usually an indicator of synovitis associated with active disease [45]. Although we found no difference in swollen joint count between fallers and non-fallers in the current study, synovitis in the small joints of the feet can be difficult to detect clinically [46]. The feet are often overlooked during consultations and it is possible that active foot disease may go unnoticed [47]. Our findings suggest that assessment of tenderness and swelling in the lower limb joints, including the feet, may be important in identifying people with RA at increased fall risk.

We found fallers to have longer RA disease duration than non-fallers. As falls are generally associated with older adults, clinicians may not identify younger people with established RA, who are at increased fall risk. Number of co-morbid conditions was also associated with increased falls risk, as observed in previous studies in RA [10, 11]. In addition, vascular disease (including stroke, ischemic heart disease, arrhythmia and peripheral vascular disease) was independently associated with a history of falling in the current study, with the odds of falling more than tripling (OR 3.2) compared to those without vascular disease. Co-morbid conditions, including cardiovascular diseases, cancer, osteoporosis and depression, are common complications of RA [5]. Therefore, an association between falls risk and co-morbid conditions, in particular vascular disease, is an important finding.

In this study, falls were not associated with older age or female sex which is in agreement with several other studies in people with RA [4, 7, 9–11, 22]. In the general population, older adults (over 65 years) and women experience significantly more falls than younger adults and men, and falls rate increases with increasing age [48]. It is possible that age related fall risk factors, such as impaired general health, co-morbid conditions, fatigue and history of prior falls, may occur in adults of all ages with RA, thus mitigating age-related differences.

Strengths of the study are the large sample size and the inclusion of a comprehensive range of validated foot and ankle measures. The study has some limitations. Firstly, the recruitment strategy involved inviting clinic patients to participate in a study of fall risk, so the frequency of falls may have been over-estimated and may not be a true representation of people with RA. Secondly, the retrospective recording of falls may be subject to recall bias [49]. There was no adjustment applied for multiple comparisons in the univariate analysis. Finally, the cross-sectional design prohibits determination of causality and it is not possible to determine the temporal nature of observed associations. Prospective analysis of this cohort is ongoing to provide more definitive conclusions regarding causes of falls in people with RA.

## Conclusions

Elevated midfoot peak plantar pressures, self-reported foot impairment and history of vascular disease are independently associated with falls in the preceding 12-months in people with RA. Assessment of foot deformity, foot function and self-reported foot impairment may therefore be of benefit when considering falls prevention strategies in people with RA.
